# Serologic responses to the PfEMP1 DBL-CIDR head structure may be a better indicator of malaria exposure than those to the DBL-α tag

**DOI:** 10.1186/s12936-019-2905-9

**Published:** 2019-08-13

**Authors:** Emily M. Stucke, Amadou Niangaly, Andrea A. Berry, Jason A. Bailey, Drissa Coulibaly, Amed Ouattara, Kirsten E. Lyke, Matthew B. Laurens, Antoine Dara, Matthew Adams, Jozelyn Pablo, Algis Jasinskas, Rie Nakajima, Albert E. Zhou, Sonia Agrawal, DeAnna J. Friedman-Klabanoff, Shannon Takala-Harrison, Bourema Kouriba, Abdoulaye K. Kone, J. Alexandra Rowe, Ogobara K. Doumbo, Philip L. Felgner, Mahamadou A. Thera, Christopher V. Plowe, Mark A. Travassos

**Affiliations:** 1 Malaria Research Program, Center for Vaccine Development and Global Health, University of Maryland School of Medicine, Baltimore, MD USA; 2 Malaria Research and Training Center, University of Science, Techniques and Technologies of Bamako, Bamako, Mali; 3 The EMMES Corporation, Rockville, MD USA; 4 Division of Infectious Diseases, Department of Medicine, University of California, Irvine, CA USA; 5 Centre for Immunity, Infection and Evolution, Institute of Immunology and Infection Research, School of Biological Sciences, University of Edinburgh, Edinburgh, UK; 6 Duke Global Health Institute, Duke University, Durham, NC USA

**Keywords:** Malaria, *Plasmodium falciparum*, *var* genes, PfEMP1, Immunity, Seroreactivity, Microarray

## Abstract

**Background:**

*Plasmodium falciparum* erythrocyte membrane protein-1 (PfEMP1) antigens play a critical role in host immune evasion. Serologic responses to these antigens have been associated with protection from clinical malaria, suggesting that antibodies to PfEMP1 antigens may contribute to natural immunity. The first N-terminal constitutive domain in a PfEMP1 is the Duffy binding-like alpha (DBL-α) domain, which contains a 300 to 400 base pair region unique to each particular protein (the DBL-α “tag”). This DBL-α tag has been used as a marker of PfEMP1 diversity and serologic responses in malaria-exposed populations. In this study, using sera from a malaria-endemic region, responses to DBL-α tags were compared to responses to the corresponding entire DBL-α domain (or “parent” domain) coupled with the succeeding cysteine-rich interdomain region (CIDR).

**Methods:**

A protein microarray populated with DBL-α tags, the parent DBL-CIDR head structures, and downstream PfEMP1 protein fragments was probed with sera from Malian children (aged 1 to 6 years) and adults from the control arms of apical membrane antigen 1 (AMA1) vaccine clinical trials before and during a malaria transmission season. Serological responses to the DBL-α tag and the DBL-CIDR head structure were measured and compared in children and adults, and throughout the season.

**Results:**

Malian serologic responses to a PfEMP1’s DBL-α tag region did not correlate with seasonal malaria exposure, or with responses to the parent DBL-CIDR head structure in either children or adults. Parent DBL-CIDR head structures were better indicators of malaria exposure.

**Conclusions:**

Larger PfEMP1 domains may be better indicators of malaria exposure than short, variable PfEMP1 fragments such as DBL-α tags. PfEMP1 head structures that include conserved sequences appear particularly well suited for study as serologic predictors of malaria exposure.

**Electronic supplementary material:**

The online version of this article (10.1186/s12936-019-2905-9) contains supplementary material, which is available to authorized users.

## Background

*Plasmodium falciparum* erythrocyte membrane protein-1 (PfEMP1) antigens are critical to parasite sequestration, and their genetic diversity likely facilitates host immune system evasion. The *var* gene family encodes PfEMP1 antigens, whose extracellular binding region is made up of Duffy-binding-like (DBL) domains interspersed with cysteine-rich interdomain regions (CIDRs). A 300–400 variable nucleotide region surrounded by semi-conserved motifs in the DBL-α domain, the first N-terminal *var* domain, acts as a unique “fingerprint” specific to individual *var*s. DBL-α “tag” sequencing facilitates analyses of both PfEMP1 genetic diversity [1, 2] and seroreactivity [3, 4].

Antibody responses to locally derived DBL-α tags in Papua New Guinea increased in magnitude and prevalence with age in children, suggesting that DBL-α tags correlate with malaria exposure and potentially the development of natural immunity to malaria [4]. In contrast, a subsequent study of seroreactivity to 3D7 strain PfEMP1 antigens found that sera from children with cerebral malaria or severe malarial anaemia did not differ in recognition of DBL-α tags compared to sera from matched uncomplicated malaria controls in Bandiagara, Mali, but did with respect to larger extracellular PfEMP1 fragments [5].

To identify accurate markers of malaria exposure, serologic responses to the DBL-α tag of a PfEMP1 and its constitutive domains were measured. Serum samples from malaria-exposed Malian children, aged 1 to 6 years, and adults were studied on a custom PfEMP1 microarray populated with DBL-α tags, parent DBL-CIDR head structures (i.e. the entire DBL-α domains encompassing the tags with the succeeding CIDR domains), and downstream PfEMP1 fragments. The hypothesis was that seroreactivity to DBL-α tags among malaria-exposed individuals increases with age and with cumulative *P. falciparum* exposure. In addition, seroreactivity to the DBL-α tag was not predicted to correlate with seroreactivity to the parent DBL-CIDR head structure, given the head structure’s additional structural components and its relative sequence conservation.

## Methods

Protein microarray construction followed a four-step process that included: (1) PCR amplification of each complete or partial *P. falciparum* open reading frame, (2) in vivo recombination cloning, (3) in vitro transcription/translation (IVTT), and (4) microarray chip printing [6]. This IVTT protein microarray platform has produced antibody responses strongly correlated with those from ELISA assays featuring purified proteins of several malaria vaccine candidate antigens [7]. The microarray included nine DBL-α tags that encode Group A, B, B/A, and C PfEMP1 antigens from the 3D7 reference genome; nine matched parent DBL-CIDR head structures; and 18 matched downstream PfEMP1 fragments. Each DBL-α tag was expressed as a single protein fragment, and the corresponding DBL-CIDR head structure was expressed as a single protein fragment.

The protein microarrays were probed with sera from 18 adults aged 18–55 years old in the control arm of a trial of an apical membrane antigen 1 (AMA1) vaccine adjuvanted to GlaxoSmithKline’s AS02A (FMP2.1/AS02A) [8] and from 35 children aged 1 to 6 years in the control arm of a Phase II AMA1 vaccine (FMP2.1/AS02A) trial [9] using published methods [6]. These serum samples were collected with respect to two malaria transmission seasons in rural Mali [adults: June 2005 (pre-malaria season) and December 2005 (post malaria season); children: May 2007 (pre-malaria season) and September 2007 (peak-malaria season)]. Sera from 11 US blood donors were used as negative controls. The data were background subtracted using the mean of the no-DNA controls, and negative fluorescence intensities were zeroed.

All participants or guardians of participants provided written informed consent, and the trial was conducted under the Declaration of Helsinki. The institutional review boards of the Faculty of Medicine, Pharmacy and Dentistry, Bamako, Mali, and the University of Maryland approved the study protocol.

### Analysis

#### Seroprevalence

Seroprevalence is the proportion of serum samples that recognized a protein fragment. A serum sample recognized a protein fragment if the fluorescence intensity was greater than two standard deviations above the malaria-naïve control mean for that fragment [10]. Group “recognition” of a protein fragment was defined as a pre-malaria transmission season mean fluorescence intensity for Malian children or adults that was greater than malaria-naïve controls, based on a two-sample Kolmogorov–Smirnov test as previously described [5, 11]. The McNemar’s test was used to determine if the proportions of serorecognized DBL-α tags and corresponding DBL-CIDR head structures were significantly discordant.

#### Seroreactivity

Change in seroreactivity for a protein fragment between the pre- and peak season for children and pre- and post-transmission season for adults was determined with a Wilcoxon signed-rank test for matched samples. Correlation between seroreactivity of DBL-α tag and DBL-CIDR head structures was measured with the Spearman correlation coefficient, *r*_*s*_. P-values were two-sided, and α = 0.05. No adjustment was made for multiple comparisons as per previous protein microarray analyses [5, 7, 12].

## Results

**Fig. 1:**
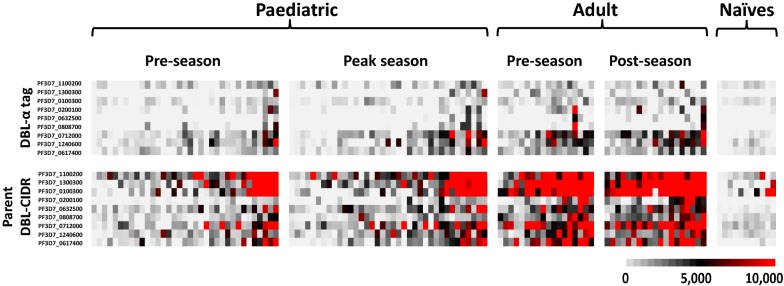
Heat map of seroreactivity to 9 PfEMP1 DBL-α tags and their parent DBL-CIDR head structures for sera from 35 Malian children, 18 adults, and 11 malaria-naïve controls, with each protein fragment separated by row, including pre-season seroreactivity and peak- or post-season seroreactivity. Each column displays the profile of one serum sample. Seroreactivities of PfEMP1 protein fragments were ordered from least to most reactive parent DBL-CIDR head structures. Individual serum samples within each subgroup were ordered from least to most seroreactive for all PfEMP1 protein fragments. Grey indicates no seroreactivity, black is low to moderate seroreactivity, and red denotes high seroreactivity to probed fragments, measured by fluorescence intensity. Seroreactivity of malaria-naïve controls was used to determine serorecognition of protein fragments by malaria exposed children and adults. PfEMP1, *Plasmodium falciparum* erythrocyte membrane protein-1; DBL, Duffy binding-like; CIDR, cysteine-rich interdomain region

Fluorescence intensities to DBL-α tags and their parent DBL-CIDR head structures were measured using sera collected from Malian children and adults and North American malaria-naïve adult controls (Fig. 1). Overall, increased serologic responses to the DBL-CIDR domains compared to their corresponding DBL-α tags were observed for both children and adults.

### Seroprevalence

**Fig. 2:**
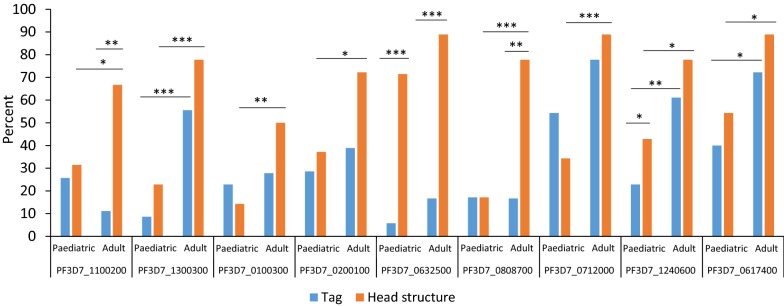
Seroprevalence of DBL-α tag fragments and parent DBL-CIDR head structures recognized at pre-season for each PfEMP1 in children and adults. Paediatric sera recognized a discordant proportion of the DBL-α tag and parent DBL-CIDR head structure for two PfEMP1s, PF3D7_0632500 and PF3D7_0317400. Adult sera recognized discordant proportions of DBL-α tag and parent DBL-CIDR head structure for three PfEMP1s, PF3D7_1100200, PF3D7_0632500, and PF3D7_0808700. (*P < 0.05, **P < 0.01; ***P < 0.001. The McNemar’s test was used to compare tags and head structures within paediatric and adults groups. For comparisons between adults and children, the Chi square test was used.) PfEMP1, *Plasmodium falciparum* erythrocyte membrane protein-1; DBL, Duffy binding-like; CIDR, cysteine-rich interdomain region

Comparing the seroprevalence of DBL-α tags and DBL-CIDR head structures, serorecognition of DBL-α tags did not consistently predict parent DBL-CIDR head structure serorecognition (Fig. 2). For two of the nine PfEMP1 antigens, a discordant proportion of paediatric sera recognized the DBL-α tags compared to the proportion recognizing the corresponding parent DBL-CIDR head structure (PF3D7_0632500: P < 0.0001, PF3D7_01240600: P = 0.04, McNemar’s test; Fig. 2). For all nine PfEMP1 antigens, a greater proportion of adult sera recognized the parent DBL-CIDR head structure compared to the proportion recognizing the corresponding DBL-α tag; however, the proportion was significantly discordant for three PfEMP1 antigens (PF3D7_1100200: P = 0.0009, PF3D7_0632500: P = 0.0008, PF3D7_0808700: P = 0.003, McNemar’s test; Fig. 2).

For three PfEMP1 antigens, paediatric and adult sera demonstrated equivalent DBL-α tag serorecognition, but the proportion of DBL-CIDR head structure serorecognition was greater in adults versus children (PF3D7_1100200: P < 0.05, PF3D7_0100300: P < 0.05, PF3D7_0200100: P < 0.05, PF3D7_0808700: P < 0.0001; PF3D7_0712000: P < 0.0001; Chi square test; Fig. 2). For PF3D7_0632500, sera from a minority of children and adults recognized the DBL-α tag, but sera from most children and adults recognized the parent DBL-CIDR domain head structure. This proportion was higher in adults compared to children for both the DBL-α tag (P < 0.0001; Chi square test) and the DBL-CIDR domain head structure (P < 0.001; Chi square test).

#### Children

**Fig. 3:**
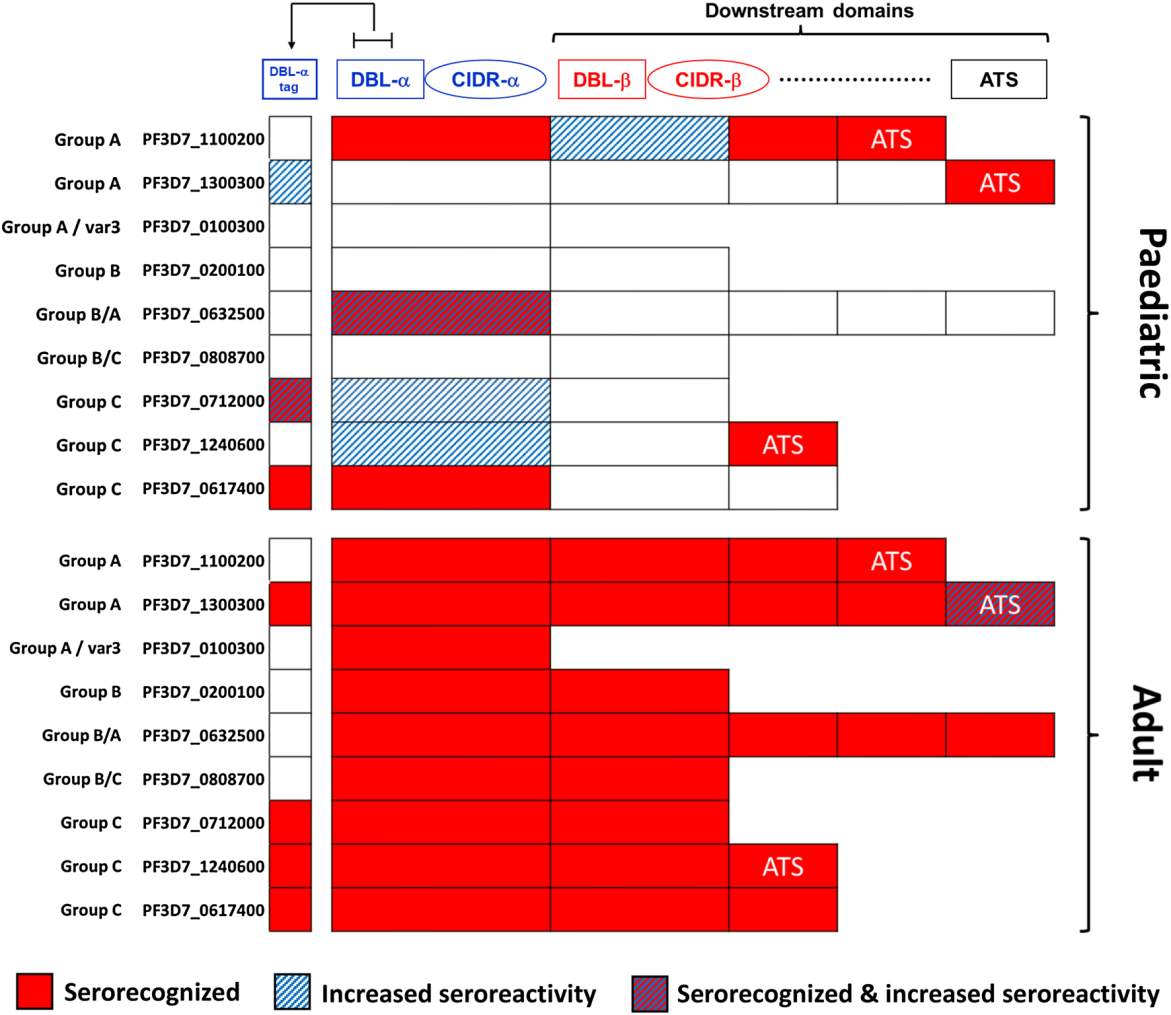
Serorecognition and seroreactivity patterns of Malian children (top) and adults (bottom) for 36 protein fragments of nine 3D7 PfEMP1 antigens. Each DBL-α tag of a PfEMP1 antigen is the first fragment shown. Group serorecognized fragments are indicated in red, while blue stripes indicate increased seroreactivity of a fragment over a malaria season from pre-season to peak season for children and from pre-season to post-season for adults. PfEMP1, *Plasmodium falciparum* erythrocyte membrane protein-1; DBL, Duffy binding-like; CIDR, cysteine-rich interdomain region; ATS, acidic terminal segment

As a group, Malian paediatric sera recognized two of nine DBL-α tags, three of nine DBL-CIDR head structures, and four of 18 downstream PfEMP1 fragments (Fig. 3). Of the three recognized parent DBL-CIDR head structures, children recognized only one corresponding DBL-α tag (from PF3D7_0617400). Paediatric sera recognized the DBL-α tag from PfEMP1 PF3D7_0712000, but neither of the larger domain fragments that represented the extracellular region. Paediatric sera recognized all three ATS fragments, but only one of 18 extracellular PfEMP1 fragments downstream of the DBL-CIDR head structure.

#### Adults

Malian adult sera recognized all nine of the parent DBL-CIDR head structures, but only four corresponding DBL-α tags (Fig. 3). Adult sera recognized all 11 downstream fragments and the two ATS fragments of the four PfEMP1 antigens with serorecognized DBL-α tags. In total, Malian adult sera recognized 4/9 DBL-α tags, 9/9 parent DBL-CIDR head structures, and 18/18 downstream PfEMP1 fragments (Fig. 3).

### Seroreactivity

#### Children

During a malaria transmission season, paediatric sera had increased seroreactivity to two DBL-α tags (PF3D7_1300300: P = 0.04 and PF3D7_0712000: P = 0.02), but only one of the corresponding parent DBL-CIDR head structures also had increased seroreactivity (PF3D7_0712000: P = 0.01; Fig. 3). Paediatric sera also had increased seroreactivity to two DBL-CIDR head structures (PF3D7_0632500: P = 0.01 and PF3D7_1240600: P = 0.01), but not to their corresponding DBL-α tags. Sera from children had increased seroreactivity during a malaria transmission season to only one downstream PfEMP1 fragment (PF3D7_1100200, P = 0.048; Wilcoxon signed-rank test).

#### Adults

For adults, seroreactivity to PfEMP1 antigens remained unchanged for all but one PfEMP1 fragment, which increased (the ATS of PF3D7_1300300: P = 0.02; Wilcoxon signed-rank test; Fig. 3) over the course of the malaria season.

#### Correlation of seroreactivity

For serum samples with serorecognition of a DBL-α tag and/or corresponding parent DBL-CIDR head structure, correlation of the reactivities to the DBL-α tag (measured by fluorescence intensity) and to the corresponding DBL-CIDR domain was determined. Reactivity to a DBL-α tag did not correlate well with reactivity to the corresponding parent DBL-CIDR head structure (Table 1). DBL-α tag seroreactivity significantly correlated with the parent DBL-CIDR head structure seroreactivity for only two PfEMP1 antigens (PF3D7_1100200: *r*_*s*_= 0.81, P = 0.007; PF3D7_0712000: *r*_*s*_= 0.52, P = 0.002; Table 1).

**Table 1:** Correlation of DBL-α tags and parent DBL-CIDR head structures for serorecognized PfEMP1 fragments

*var* group	PfEMP1	No. of serum samples with serorecognition of the fragment	*r*_*s*_	*P* value
A	PF3D7_1100200	11	0.81	0.007
	PF3D7_1300300	13	0.42	0.15
A/var3	PF3D7_0100300	13	0.36	0.23
B	PF3D7_0200100	17	0.33	0.19
B/A	PF3D7_0632500	5	− 0.30	0.68
B/C	PF3D7_0808700	9	0.58	0.11
C	PF3D7_0712000	33	0.52	0.002
	PF3D7_1240600	19	− 0.10	0.68
	PF3D7_0617400	27	0.34	0.09

*PfEMP1 Plasmodium falciparum* membrane protein-1, *DBL* Duffy binding-like, *CIDR* cysteine-rich interdomain region, *r*_*s*_ Spearman correlation coefficient

## Discussion

Serologic responses to a PfEMP1’s DBL-α tag region did not correlate with seasonal malaria exposure or with responses to the parent DBL-CIDR head structure in either adults or children from an area of Mali with intense seasonal exposure to *P. falciparum* malaria. Parent DBL-CIDR head structures were better indicators of malaria exposure. Serologic prediction of malaria exposure may be better estimated by using larger PfEMP1 fragments, such as head structures that include conserved regions, as targets for serological testing.

DBL-α tags have been used as a unique “fingerprint” reflecting PfEMP1 diversity in gene expression, serologic, and population genomics studies [4, 13–15]. These DBL-α tag sequences serve as a useful means of PfEMP1 classification, because each PfEMP1 contains only one DBL-α domain and most PfEMP1 antigens include a DBL-α domain. The DBL-α tag consists of a 300 to 400 base pair variable region flanked by conserved motifs that provide targets for degenerate primers [16, 17]. The findings here suggest that such a unique sequence tag may not perform well as a sensitive marker for malaria exposure, potentially due to the diverse nature of the protein fragment. Seroreactivity to the parent DBL-CIDR head structure containing more conserved sequences better identified age and seasonal malaria exposure.

These findings contrast with results from a study done in Papua New Guinea that found that serologic responses to DBL-α tags revealed differences in malaria exposure. One hundred twenty-three DBL-α tag sequences from Papua New Guinea populated a protein microarray that was used to seroprofile Papua New Guinea adults and children, revealing that children’s antibody responses peaked between four and 15 years of age [4]. In this Papua New Guinea population, DBL-α tags served as a marker for age-related exposure to malaria. Of note, DBL-α tags from Papua New Guinea are less diverse than DBL-α tags from global populations. In a population genomic analysis of DBL-α tags from both Papua New Guinea and other malaria-endemic regions, the number of unique DBL-α tags plateaued with 30 Papua New Guinea isolates examined, but no such plateau was evident with more than 1000 DBL-α tag sequences from 59 global isolates [2]. Serologic responses to Papua New Guinea DBL-α tags may therefore be more informative than responses to DBL-α tags in locations with more genetically diverse DBL-α tags. In the current study, age-related Malian serologic responses to DBL-α tags were not observed. However, we evaluated fragments from nine PfEMP1 antigens compared to the 123 evaluated from Papua New Guinea. It is possible that with a large enough pool of DBL-α tags on a microarray and a larger sample size, differences in age-related exposure may be discerned.

Antibody responses to particular PfEMP1 domains have been associated with protection from clinical malaria in several studies [7, 18–20], and may play a role in protection from severe malaria disease. A recent study of antibody responses in Malian children with severe malarial anaemia and/or cerebral malaria found gaps in immunity to particular PfEMP1 antigens compared to uncomplicated malaria controls [5]. Sera from children with severe malaria recognized fewer PfEMP1 antigens and reacted less intensely to particular PfEMP1 fragments compared to controls with uncomplicated malaria. Immunologic gaps included several DBL-CIDR domains, but only two associated DBL-α tags. In fact, immunologic gaps to particular DBL-CIDR head structures did not predict a gap to a particular DBL-α tag, and vice versa. This is consistent with the findings here that serologic responses to DBL-α tags were not as predictive as markers of malaria exposure compared to responses to larger PfEMP1 fragments such as head structures.

Earlier microarray studies have found that more conserved PfEMP1 fragments are markers of malaria exposure. In a study of Malian children and adults followed during the malaria season, the most differentially seroreactive fragments in adults compared to children contained the intracellular ATS domain, which is more conserved than extracellular domains [11, 21]. Similarly, in this study, paediatric sera recognized all three of the ATS domains. In Malian *P. falciparum* genomes, DBL-α and DBL-β domains have more sequence conservation than other DBL domains [22]. Here, children recognized head structures containing DBL-α and DBL-β domains while other extracellular PfEMP1 domains with lower sequence conservation remained unrecognized. Taken together, these findings suggest that when using PfEMP1 antigens as microarray markers of malaria exposure, the most conserved portions may be the most informative for malaria exposure. Such ordered serorecognition of PfEMP1 domains based on sequence conservation may also underlie the order of PfEMP1 domain recognition identified in Tanzanian children during the first 2 years of life [23, 24].

This study had some limitations. The examined DBL-α domains were from the reference strain 3D7, not from circulating Malian parasites in the population. In spite of this, individuals had seroreactivity to head structures and to the downstream PfEMP1 fragments, particularly Malian adults, who as a group recognized all DBL-CIDR head structures and downstream fragments. This suggests that the PfEMP1 antigens from 3D7 may represent the much larger circulating PfEMP1 population in Mali to some degree. Another limitation is the limited DBL-α tag population studied: DBL-α tags from nine 3D7 PfEMP1 antigens were examined, which represents only a small subset of the 60 3D7 PfEMP1 antigens, although at least one member of each PfEMP1 subgroup was included. This limited representation of PfEMP1 antigens could be another possible explanation for the limited serorecognition by children. It is possible that DBL-α tags from other 3D7 PfEMP1 antigens could have elicited more serorecognition in Malian children, but were not represented on the microarray. Whereas proteins expressed on this microarray platform have been shown to induce antibody responses similar to those from purified proteins, these proteins are produced with an *Escherichia coli* system and lack post-translational modifications such as glycosylation [7]. Correct folding of the PfEMP1 proteins produced by the *Escherichia coli* expression system has not been confirmed.

To further examine the role of conserved PfEMP1 domains in inducing serologic responses, peptide microarrays are a potentially useful tool to identify reactive epitopes. Such an approach may provide insight distinct from protein microarrays, where entire domains are included and may elicit differential seroreactivity. A protein microarray does not permit identification of the specific roles of either more conserved or more diverse regions within a protein fragment. Peptide arrays have already been used as a tool for studying epitopes in variant surface antigen proteins in *Plasmodium falciparum*. In particular, they have recently been used to pinpoint epitopes targeted by antibodies in a DBL-α domain that mediates rosetting [25] and epitopes within particular domains of STEVOR and RIFIN antigens reflecting malaria exposure [26]. This approach could be used to identify epitopes targeted by protective PfEMP1 antibodies and thereby inform vaccine or therapeutics design.

## Conclusions

Malian serologic responses to a PfEMP1’s DBL-α tag, a semi-conserved short fragment of the DBL-α domain, did not correlate with seasonal malaria exposure or responses to the parent DBL-CIDR head structure in either adults or children. Larger, more conserved PfEMP1 domains may be better indicators of malaria exposure than short, variable PfEMP1 fragments such as DBL-α tags. PfEMP1 head structures that include conserved sequences are thus well suited for study as serologic predictors of malaria exposure on protein microarrays. Further support for this finding would include testing a broader panel of PfEMP1 antigens, including those from non-3D7 reference strains and from endemic parasite genomes.

## Additional file

**Additional file 1.** Sequences, classifications, and domains of the PfEMP1 antigens used to populate the custom protein microarray.

## Data Availability

Sequences used in this study are available in Additional file 1: Table S1.
